# Post-migration trajectories and psychopathological vulnerability

**DOI:** 10.1192/j.eurpsy.2023.138

**Published:** 2023-07-19

**Authors:** I. Tarricone, Ilaria Tarricone, Giuseppe D’Andrea, Hannah E Jongsma, Sarah Tosato, Charlotte Gayer-Anderson, Simona A. Stilo, Federico Suprani, Conrad Iyegbe, Els van der Ven, Diego Quattrone, Marta di Forti, Eva Velthorst, Paulo Rossi Menezes, Celso Arango, Mara Parel

**Affiliations:** Department of Medical and Surgical Sciences, Bologna Univerisity, Bologna, Italy

## Abstract

**Abstract: Background:**

Psychosis rates are higher among some migrant groups. We hypothesized that psychosis in migrants is associated with cumulative social disadvantage during different phases of migration.

**Methods:**

We used data from the EUropean Network of National Schizophrenia Networks studying Gene-Environment Interactions (EU-GEI) case-control study. We defined a set of 3 indicators of social disadvantage for each phase: pre-migration, migration, and post-migration.

**Results:**

249 cases and 219 controls were assessed. Pre-migration (OR 1.61, 95%CI 1.06-2.44, p=0.027) and postmigration social disadvantages (OR 1.89, 95%CI 1.02-3.51, p=0.044), along with expectations/achievements mismatch (OR 1.14, 95%CI 1.03-1.26, p=0.014) were all significantly associated with psychosis. We found a dose-response effect between number of adversities across all phases and odds of psychosis (≥6: OR 14.09, 95%CI 2.06-96-47, p=0.007).

**Conclusions:**

The cumulative effect of social disadvantages before, during and after migration was associated with increased odds of psychosis in migrants, independently of ethnicity or length of stay in the country of arrival. Public health initiatives that address the social disadvantages that many migrants face during the whole migration process and post-migration psychological support may be reduce the excess of psychosis in migrants.

**Disclosure of Interest:**

None Declared

